# Automatic Impervious Surface Area Detection Using Image Texture Analysis and Neural Computing Models with Advanced Optimizers

**DOI:** 10.1155/2021/8820116

**Published:** 2021-02-16

**Authors:** Nhat-Duc Hoang

**Affiliations:** ^1^Institute of Research and Development, Duy Tan University, Da Nang 550000, Vietnam; ^2^Faculty of Civil Engineering, Duy Tan University, Da Nang 550000, Vietnam

## Abstract

Up-to-date information regarding impervious surface is valuable for urban planning and management. The objective of this study is to develop neural computing models used for automatic impervious surface area detection at a regional scale. To achieve this task, advanced optimizers of adaptive moment estimation (Adam), a variation of Adam called Adamax, Nesterov-accelerated adaptive moment estimation (Nadam), Adam with decoupled weight decay (AdamW), and a new exponential moving average variant (AMSGrad) are used to train the artificial neural network models employed for impervious surface detection. These advanced optimizers are benchmarked with the conventional gradient descent with momentum (GDM). Remotely sensed images collected from Sentinel-2 satellite for the study area of Da Nang city (Vietnam) are used to construct and verify the proposed approach. Moreover, texture descriptors including statistical measurements of color channels and binary gradient contour are employed to extract useful features for the neural computing model-based pattern recognition. Experimental result supported by statistical test points out that the Nadam optimizer-based neural computing model has achieved the most desired predictive accuracy for the data collected in the studied region with classification accuracy rate of 97.331%, precision = 0.961, recall = 0.984, negative predictive value = 0.985, and F1 score = 0.972. Therefore, the model developed in this study can be a helpful tool for decision-makers in the task of urban land-use planning and management.

## 1. Introduction

Urban impervious surface, developed by anthropogenic activities, is one of the most crucial land cover forms. The impenetrable surface areas consist of buildings, roads, parking lots, sidewalks, pavements, and many others. These surfaces prevent the absorption of water into the soil. Previous works have pointed out the impact of impervious surface areas on water quality and the frequency/intensity of downstream runoff [1–5]. Therefore, they have been identified as a key indicator used in evaluating urbanization influences on surrounding natural environment and ecosystem [6].

Due to such reasons, up-to-date information regarding impervious surface is of paramount importance for supporting urban land management/planning, detection of unplanned built-up areas, study of regional land-use pattern, and ecosystem monitoring [5, 7–10]. In developing countries including Vietnam, the conventional approach for obtaining such information is field survey. Nevertheless, this approach is very time-consuming and requires considerable effort in data collection, processing, and storing. Therefore, a quick and cost-effective method for substituting this conventional approach is a practical need for municipal land-use managers.

In recent years, remote sensing technology and processing of satellite images have been increasingly applied to tackle various challenging problems in a wide span of domains including agriculture [11–13], natural hazard prevention [14–17], civil engineering [18–20], and environmental engineering [21–23].

Following this trend of research, scholars and practitioners have increasingly relied on remote sensing and geographic information system (GIS) technologies to improve the productivity and accuracy of the impervious surface detection task [24, 25]. These technologies have been proven to be viable tools for surveying urban landscapes which are rapidly changing and providing timely information regarding urban growth [26–29]. Based on remotely sensed images, statistical and machine learning models can be constructed for automatic impervious surface extraction [8].

Lo [30] developed a computer-based model for analyzing remote sensing data obtained from Landsat image; this model only relied on spectral information of image pixels to derive land form categories. Zha et al. [29] performed built-up areas mapping with the utilization of normalized difference vegetation index and normalized difference built-up index; the proposed model analyzed Landsat Thematic Mapper images and achieved an accuracy of 92.6%. A multivariate statistical analysis approach has been put forward in [28] for characterizing urban growth; this approach could reduce the modeling error to less than 10%. Yang et al. [5] employed a combination of Landsat ETM+ and high-resolution imagery to construct a decision tree-based impervious surface mapping. Multilayer perceptron neural network and support vector machine have been used in [6] to classify image samples obtained from Landsat-5 TM Imager.

Zhang et al. [31] integrated spectral information and multivariate texture to extract numerical features from remotely sensed image; the one-class support vector machine is then used for pattern classification. Zhang et al. [32] investigated the capability of random forest approach for impervious surface estimation with a combined utilization of synthetic aperture radar and optical remote sensing images. A backpropagation neural network has been constructed by Patel and Mukherjee [33] to extract the impervious features using Landsat Thematic Mapper data. Son et al. [34] introduced an impervious surface fraction algorithm (ISFA) for automatic impervious surface extraction; this algorithm is applied with Landsat data and attains an accuracy of 92.8%. Gupta et al. [7] compared the performances of supervised maximum likelihood algorithms, index-based classification, and neural classification and points out that the neural classification model achieves the most desired outcome.

It can be seen from the literature that most of the previous works have employed the medium resolution open-source image dataset such as Landsat Thematic Mapper to extract impervious surface [35]. Because of the complex texture of urban landscape, these coarse resolution images feature certain limitations on impervious surface mapping. Xu et al. [36] investigated the use of the 10 m resolution Sentinel-2A dataset for impervious area extraction and pointed out the superiority of high-resolution data over the conventional 30 m resolution Landsat dataset. Misra et al. [8] attempted to employ high-resolution image obtained from Sentinel-2 to improve the quality of impervious surface detection result; the authors rely on spectral angle mapper, support vector machine, and neural network to carry out pattern recognition task.

Among the machine learning approaches employed in remote sensing and GIS field, neural computing models have been extensively employed and remain effective tools for recognizing patterns in remotely sensed images [37–44]. It is because neural computational models with their capability of universal function approximator are capable of learning and recognizing complex patterns [45]. Nevertheless, the employed neural computing approaches have mainly relied on the conventional gradient descent for model training [7, 8, 37, 46]. Although this conventional training method can help to attain acceptable results in many application cases, it also suffers from slow convergence rate and trapping in local optimal [47]. These facts definitely reduce the generalization and accuracy of prediction models constructed by neural computational approaches. Therefore, there is a pressing need to investigate and apply advanced training algorithms to mitigate the disadvantages of the conventional gradient descent.

In recent years, various advanced gradient-based optimization algorithms have been proposed and used for training neural computing models. However, few research works have investigated these state-of-the-art algorithms in constructing neural computing models used for remote sensing-based impervious surface detection. Therefore, this study is an attempt to fill this gap in the current literature. The advanced optimizers of adaptive moment estimation (Adam) [48], a variation of Adam called Adamax [48], Nesterov-accelerated adaptive moment estimation (Nadam) [49], Adam with decoupled weight decay (AdamW) [50], and a new exponential moving average variant (AMSGrad) [51] are employed for automatic impervious surface extraction.

In addition, Da Nang city (Vietnam) has been selected as the study area. Image texture analysis technique including statistical measurements of color channels [52] and binary gradient contour [53] are used to extract useful features from remotely sensed images obtained from Sentinel-2 satellite. The extracted features are then employed by neural computing models for automatic impervious surface detection in the study area. Therefore, one major contribution of the current study is to establish an advanced hybridization of machine learning and image processing used for constructing an impervious surface map for the study area of Da Nang city.

The subsequent sections of the article are organized as follows: the research methodology is reviewed in the second section. The proposed neural computing model trained by the aforementioned advanced optimizers used for impervious surface area detection is presented in the next section, followed by the fourth section which reports experimental results. Several concluding remarks on the current study are stated in the final section.

## 2. Research Methodology

### 2.1. General Description of the Study Area

**Figure 1:**
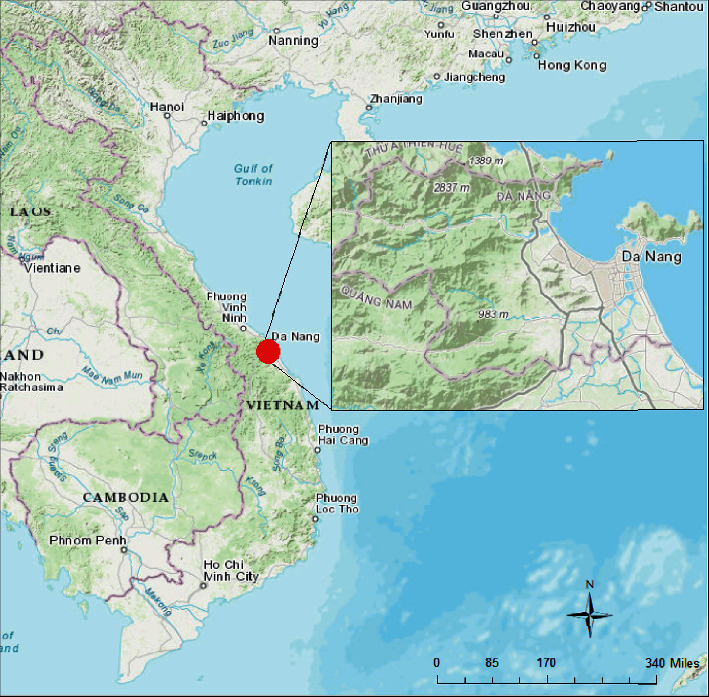
The study area.

Da Nang city is located in the Central Vietnam (refer to Figure 1). Its latitude is between 15°15′20″N and 16°14′10″N; its longitude is from 107°18′30″E to 108°20′00″E [54]. It is a port city located on the coast of the East Sea. In 2015, Da Nang had a population of 1,046,876 and an area of 1,285.4 km^2^ [55]. This city is divided into 8 districts: 6 urban districts and 2 rural districts [56].

Da Nang is ranked as the fourth largest city by population in Vietnam and serves as an economic base in the service and industrial sectors in Central Vietnam. Due to such reasons, the population of this city is rising rapidly from approximately 673,000 in 1997 to about 1 million in 2014 [57]. This population growth leads to a significant urban expansion. Therefore, Da Nang city is selected as the study area in this article.

### 2.2. The Image Data Used

**Figure 2:**
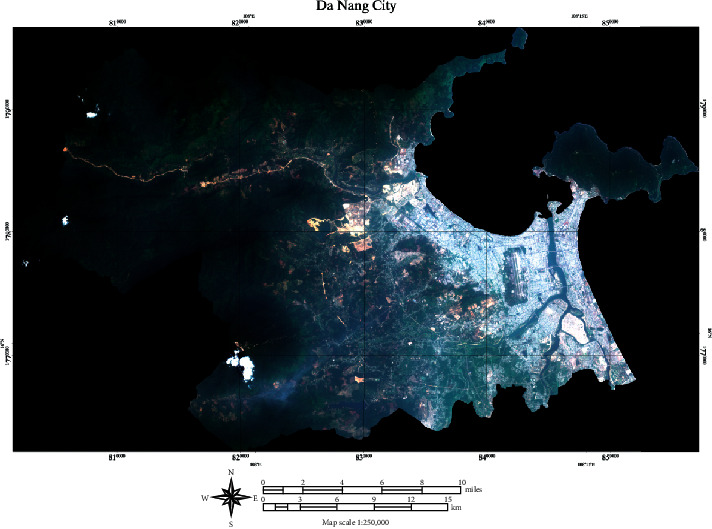
Color composite of Sentinel-2 bands 4 (red), 3 (green), and 2 (blue) of the study area (Da Nang city, Vietnam).

The image data obtained from the Sentinel-2 on March 13, 2020, is used in this study to perform impervious surface extraction. The bands of 4 (red), 3 (green), and 2 (blue) with spatial resolution of 10 m are selected to compose the image of the study area. The size of each image file (i.e., bands 2, 3, and 4) is 235,484 KB. A full-scene map of Da Nang city (5559 × 3444 pixels) is presented in Figure 2. It is noted that these Sentinel-2's bands have been opened in Sentinel Application Platform (SNAP) software package [58]. The original Sentinel-2's bands obtained from USGS [59] are converted to TIF format using the geometric operation of resampling supported by the SNAP software package. For more details of the SNAP software documentation, readers are guided to articles provided in [60]. Moreover, the used map projection of the obtained images is Universal Transverse Mercator (UTM) within Zone 48N–Datum World Geodetic System (WGS) 84.

**Figure 3:**
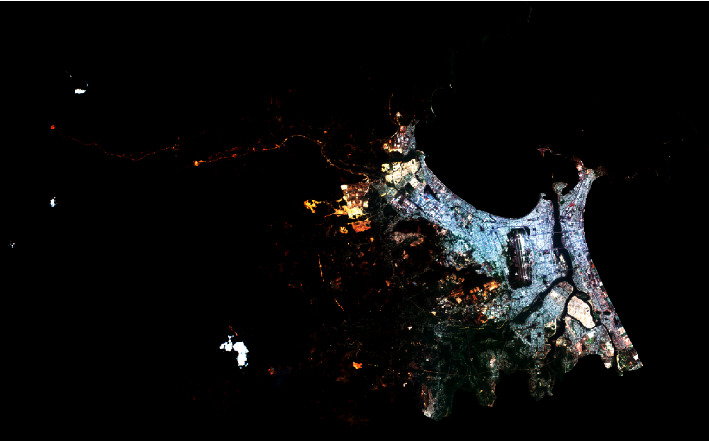
The map of the study area enhanced by histogram equalization.

Based on the original composed image, the contrast enhancement technique of histogram equalization (refer to Figure 3) is employed to create a better image for subsequent analysis. The purpose of histogram equalization is to construct an image with equally distributed brightness levels [61]. This image processing technique meliorates the global contrast of the original image and highlights the image texture. In addition, to facilitate the process of impervious surface detection, pixels at mountainous regions covered by cloud and large beach areas are cast out by masking operation [62]. In addition, the normalized difference vegetation index (NDVI) [63] is computed to remove large water bodies from the study area. Via experimentation with the collected image, pixels belonging to large water bodies are associated with negative NDVI values and can be effectively excluded. The NDVI computation requires the band 4 (red) and band 8 (near-infrared band); it is obtained via the following equation:

(1) $$\begin{array}{c} \text{NDVI}=\frac{\text{NIR}-B4}{\text{NIR}+B4}, \end{array}$$

where NIR and B4 denote a near-infrared band and band 4, respectively.

### 2.3. Image Texture Analysis

#### 2.3.1. Statistical Measurements of Image Bands

For the purpose of impervious surface detection, the statistical measurements of bands 4 (red), 3 (green), and 2 (blue) are employed in this study. It is noted that this research performs impervious surface detection for each image patch of 10 × 10 pixels. Thus, to derive statistical measurements, the first-order histogram of an image patch *S* denoted as *P* (*I*) is computed as follows [52, 64]:

(2) $$\begin{array}{c} P_{b}\left(I\right)=\frac{N_{I,b}}{\text{PN}}, \end{array}$$

where *b* denotes a band index, *N*_*I*,*b*_ is the number of pixels having the value of *I*, and PN denotes the number of pixels within an image patch.

Accordingly, the mean (*μ*_*b*_), standard deviation (*σ*_*b*_), skewness (*S*_*b*_), kurtosis (*K*_*b*_), entropy (*E*_*b*_), and range (*R*_*b*_) are computed as follows [52, 64]:

(3) $$\begin{array}{c} \mu_{b}=\sum_{i=0}^{\text{NL}-1}I_{i,b}\times P_{b}\left(I\right),\\ \sigma_{b}=\sqrt{\sum_{i=0}^{\text{NL}-1}{\left(I_{i,b}-\mu_{b}\right)}^{2}\times P_{b}\left(I\right)},\\ S_{b}=\frac{\sum_{i=0}^{\text{NL}-1}{\left(I_{i,b}-\mu_{b}\right)}^{3}\times P_{b}\left(I\right)}{\sigma_{b}^{3}},\\ K_{b}=\frac{\sum_{i=0}^{\text{NL}-1}{\left(I_{i,b}-\mu_{b}\right)}^{4}\times P_{b}\left(I\right)}{\sigma_{b}^{4}},\\ E_{b}=-\sum_{i=0}^{\text{NL}-1}P_{b}\left(I\right)\times {\text{log}}_{2}\left(P_{b}\left(I\right)\right),\\ R_{b}=\text{Max}\left(I_{b}\right)-\text{Min}\left(I_{b}\right), \end{array}$$

where *I*_*i,b*_ = 0, 1, 2,…, 255. For 8-bit image, *NL* = 256 represents the number of discrete intensity values.

#### 2.3.2. Binary Gradient Contour (BGC) for Texture Discrimination

Texture is a crucial tool for visual perception in computer vision. Since image patches of impervious surface and pervious surface can have different properties of coarseness, roughness, directionality, contrast, and regularity, using texture analysis can be helpful to delineate them. This study employs the BGC technique [53] for the task of texture discrimination. The BGC combines the analyses of local structures and occurrences to derive texture features. Notably, this method has the advantages of low computational expense and invariant to monotonic illumination changes [65]. It also possesses good discriminative powers demonstrated in previous studies [53, 66].

Essentially, the BGC carries out a pairwise comparison of adjacent pixels located in one or more closed paths along the periphery of a neighborhood of the size 3 × 3 pixels [65]. Fernandez et al. [53] put forward three versions of BGC which are the single-loop (BGC_1_), double-loop (BGC_2_), and triple-loop (BGC_3_) descriptors. To ease the description of these descriptors, a square image patch *S*_*m,n*_ is denoted as follows:

(4) $$\begin{array}{c} S=\left[\begin{array}{ccc} I_{m-1,n-1} & I_{m-1,n} & I_{m-1,n+1}\\ I_{m,n-1} & I_{m,n} & I_{m,n+1}\\ I_{m+1,n-1} & I_{m+1,n} & I_{m+1,n+1} \end{array}\right], \end{array}$$

where *I*_*m,n*_ denotes the central pixel.

Based on the definition of *S*, the single-, double-, and triple-loop BGC formulas are expressed as follows [53]:

(5) $$\begin{array}{c} {\text{BGC}}_{1}=\sum_{n=0}^{7}\lambda \left(I_{n}-I_{\left(n+1\right)\text{mod}8}\right)\times 2^{n}-1,\\ {\text{BGC}}_{2}=15\times \sum_{n=0}^{3}\lambda \left(I_{2n\text{mod}8}-I_{2\left(n+1\right)\text{mod}8}\right)\times 2^{n}+\sum_{n=0}^{3}\lambda \left(I_{2n+1}-I_{\left(2n+3\right)\text{mod}8}\right)\times 2^{n}-16,\\ {\text{BGC}}_{3}=\sum_{n=0}^{7}\lambda \left(I_{3n\text{mod}8}-I_{3\left(n+1\right)\text{mod}8}\right)\times 2^{n}-1,\;\text{where }\lambda \left(x\right)=\left\{\begin{array}{cc} 1, & \text{if }x\geq 0,\\ 0, & \text{if }x<0. \end{array}\right. \end{array}$$

### 2.4. Artificial Neural Network (ANN) for Pattern Classification

**Figure 4:**
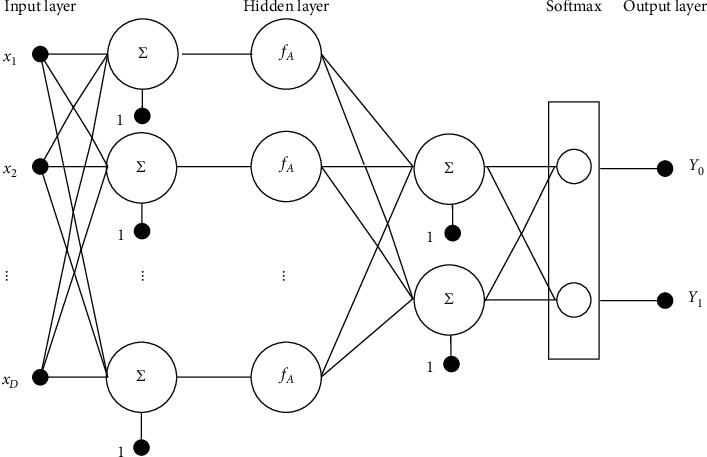
General structure of the employed ANN model used for two-class pattern recognition.

A general structure of an ANN for two-class pattern recognition tasks is presented in Figure 4. This model typically contains an input layer, a hidden layer, and an output layer [67]. In this study, the input layer receives signals in the form of texture information. The hidden layer consists of *M* neurons which process the texture information to yield the input of the softmax activation function. The usual activation function used by the neurons in the hidden layer is the log-sigmoid function. The softmax activation function converts its input signals to class probabilities within the range of 0 and 1. It is noted that interactions among neurons are expressed by connection weights. These sophisticated interactions permit the overall neural computing model to learn and infer complex mapping relationships [68].

The knowledge learnt by a neural computing model is stored in matrices of connection weights. Herein, *W*_L0L1_ denotes the matrix of connection weights between the input and hidden layer. *W*_L1L2_ represents that between the hidden and the softmax layer. Let *X* ∈ *R*^*D*^ denote the matrix of input feature. The computation process of an ANN model can be compactly defined as follows:

(6) $$\begin{array}{c} f\left(X\right)=\delta \left[b_{1}+W_{L1L2}\times \left(f_{A}\left(b_{0}+W_{L0L1}\times X\right)\right)\right], \end{array}$$

where *b*_0_ and *b*_1_ denote two bias vectors of the input and hidden layers, respectively, *f*_*A*_ represents the activation function, and *δ* represents the softmax activation function.

The softmax activation function is given by

(7) $$\begin{array}{c} \delta \left(z\right)=\frac{\text{exp}\left(z_{i}\right)}{\sum_{i=0}^{\text{CN}-1}\text{exp}\left(z_{i}\right)}, \end{array}$$

where *CN* = 2 denotes the number of output classes.

### 2.5. The Employed Network Training Methods

#### 2.5.1. The Network's Cost Function

To construct a neural computing model used for impervious surface detection, its model parameters must be identified. Herein, given a set of training data samples, the network parameters including the two matrices of *W*_L0L1_ and *W*_L1L2_ can be adapted via the framework of error backpropagation [69, 70] with mini-batch mode [71]. For the task of data classification, the cross entropy cost function is often used as the objective function (*E*) for training a neural computing model [72]. The cross-entropy function is given by

(8) $$\begin{array}{c} E=-\frac{1}{N_{d}}\sum_{n=1}^{N_{d}}T\text{ln}\left(Y\right)+\left(1-T\right)\text{ln}\left(1-Y\right), \end{array}$$

where *N*_*d*_ denotes the number of data samples; *T* and *Y* represent the actual and predicted class labels, respectively.

#### 2.5.2. The Network's Optimizers

*Gradient Descent with Momentum (GDM)*. The conventional method of gradient descent with momentum (GDM) is widely employed for training neural networks and is used as the benchmark method in this study. Via the GDM, the weights of a neural computing model are adapted as follows:

(9) $$\begin{array}{c} w_{t+1}=w_{t}-\alpha_{L}\times \frac{\text{d}E}{\text{d}w_{t}}+\lambda_{M}\times w_{t}, \end{array}$$

where *w*_*t*_ and *w*_*t*+1_ are the previous and updated network weights, *E* represents the objective function, and *α*_*L*_ and *λ*_*M*_ are the learning rate and the momentum term, respectively. *Adaptive Moment Estimation (Adam)*. The Adam, introduced by Yoshua and Yann [48], can be considered as a general algorithm for first-order gradient-based optimization of stochastic objective functions. One notable advantage of this optimizer is that it is capable of adaptively fine-tuning the learning rate parameter during the training process. The Adam relies on information obtained from the average of the second moments of the gradients. This optimizer also utilizes an exponentially decaying average of past gradients. In addition, this optimizer requires an initial setting of three hyperparameters: the step size *α* and the two exponential decay rates (*β*_1_ = 0.9 and *β*_2_ = 0.9999). When the gradient of model parameters is computed, the optimized parameters of a neural computing model are adapted via [48]

(10) $$\begin{array}{c} w_{t}=w_{t-1}-\alpha \times \frac{{\hat{m}}_{t}}{\sqrt{v_{t}}+\varepsilon }, \end{array}$$

where ${\hat{m}}_{t}$ and ${\hat{v}}_{t}$ denote the bias-corrected first moment estimate and the bias-corrected second raw moment estimate, respectively. *Adamax*. The Adamax [48] is a variant of the Adam in which the update rule for model weights is to scale their gradients inversely proportional to a *L*^*p*^ norm of their current and previous gradients. The neural network's weights are updated as follows:

(11) $$\begin{array}{c} w_{t}=w_{t-1}-\frac{\alpha }{1-\beta_{1}^{t}}\times \frac{m_{t}}{\text{max}\left(\beta_{2}u_{t-1},\left|g_{t}\right|\right)}, \end{array}$$

where *u*_*t*_ = 0 at *t* = 0; *u*_*t*_ represents the biased second raw moment estimate. *Nesterov-Accelerated Adaptive Moment Estimation (Nadam).* The Nadam optimizer, described in [49], attempts to incorporate Nesterov-accelerated adaptive moment estimation into the Adam. The major advantage of this integrated approach is that the employed adaptive moment estimation helps to perform highly accurate step in the gradient direction via updates of model parameters with the momentum step before the computation of the gradient [73]. The update rule of the Nadam is stated as follows [49, 73]:

(12) $$\begin{array}{c} w_{t}=w_{t-1}-\alpha \times \frac{{\bar{m}}_{t}}{\sqrt{{\hat{v}}_{t}}+\varepsilon }, \end{array}$$

where

(13) $$\begin{array}{c} {\bar{m}}_{t}=\left(1-\beta_{1,t}\right){\hat{g}}_{t}+\beta_{1,t+1}{\hat{m}}_{t},\\ {\hat{m}}_{t}=\frac{m_{t}}{1-\prod_{i=1}^{t+1}\beta_{1i}},\\ {\hat{g}}_{t}=\frac{g_{t}}{1-\prod_{i=1}^{t+1}\beta_{1i}}. \end{array}$$

*Adam with Decoupled Weight Decay (AdamW)*. The AdamW [50] optimizer integrates weight decay into the original Adam. The weight decay is a widely used approach for regularizing the network weights. It is because large weights may lead to an overfitted model. Accordingly, the update rule of the AdamW algorithm is given by

(14) $$\begin{array}{c} w_{t}=w_{t-1}-\alpha \times \left(\frac{{\hat{m}}_{t}}{\sqrt{{\hat{v}}_{t}}+\varepsilon }+\lambda w_{t-1}\right), \end{array}$$

where *λ* denotes a hyperparameter. *A New Exponential Moving Average Variant (AMSGrad)*. The AMSGrad optimizer [51] attempts to improve the convergence of the Adam optimizer by the employment of long-term memory of past gradient. To avoid poor convergence and trapping in local optima, Reddi et al. [51] argues that the maximum of past squared gradients *v*_*t*_ should be used for parameter update instead of the exponential average employed by the Adam optimizer. The following equation is used to update the neural network's parameter:

(15) $$\begin{array}{c} w_{t}=w_{t-1}-\alpha \times \frac{m_{t}}{\sqrt{{\hat{v}}_{t}}+\varepsilon }, \end{array}$$

where ${\hat{v}}_{t}$ denotes the updated bias-corrected 2^nd^ raw moment estimate.

## 3. The Proposed Neural Computing Model with Advanced Optimizers for Automatic Impervious Surface Detection

**Figure 5:**
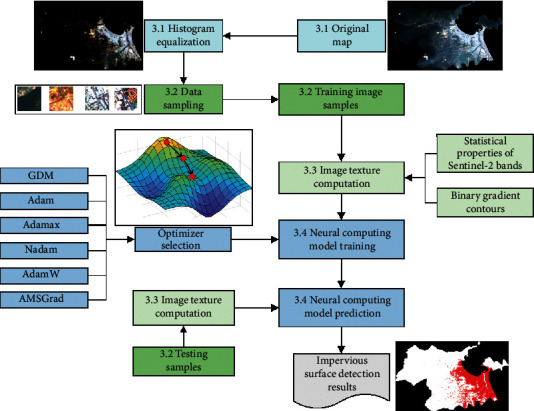
The proposed neural computing model for impervious surface detection.

This section of the article presents the general description of the proposed neural computational method employed for automatic impervious surface detection. The proposed model is an integration of image texture analysis, neural network-based pattern recognition, and advanced optimizers used for neural network training. An overview of the data processing and the training phase of the proposed neural computing model used for impervious surface detection is demonstrated in Algorithm 1. The general structure of the newly developed model is presented in Figure 5. It is noted that the proposed neural computational model used for impervious surface detection has been developed in Visual C#.NET environment (Framework 4.6.2) and performed with the ASUS FX705GE-EW165T (Core i7 8750H, 8 GB Ram, 256 GB solid-state drive).

The model operation can be divided into four steps:

- Data preprocessing
- Image data sampling
- Image texture computation
- Neural computing model training and prediction

### 3.1. Data Preprocessing

In this step, the original Sentinel-2's bands are opened in the SNAP software package and converted to TIFF format. The image process technique of histogram equalization is employed to enhance the contrast of the original image. As mentioned earlier, the NDVI is also calculated using the obtained bands to cast out large water bodies from the study area.

### 3.2. Image Data Sampling

**Figure 6:**
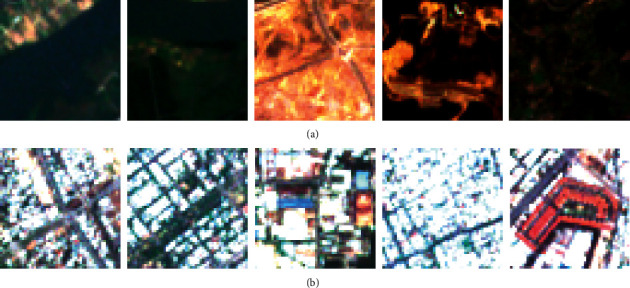
Demonstration of the collected image samples: (a) pervious class and (b) impervious class.

To establish the neural network model for automatic impervious surface area detection, it is required to prepare a training dataset with assigned ground truth labels. This study has sampled pervious and impervious areas within the map of the study area (refer to Figure 6). Each sample with the size of 100 × 100 pixels is used to create nonoverlapped image patches with the size of 10 × 10 pixels. In total, there are 3,000 image patches that are generated from image samples. To ensure a balanced dataset, the numbers of the negative (pervious surface) and positive (impervious surface) samples are both 1,500. Based on these image patches, the image texture computation methods can be carried out to extract useful feature for the pattern recognition phase.

### 3.3. Image Texture Computation

**Figure 7:**
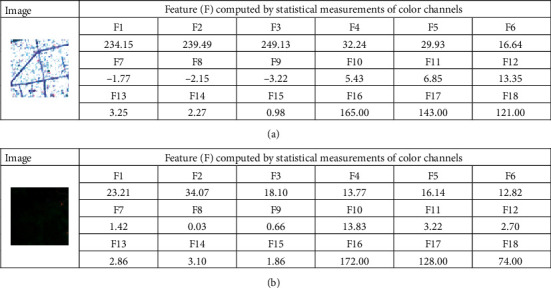
Illustration of features computed by statistical measurements of color channels: (a) an impervious surface and (b) a pervious surface.

Using image samples generated from the previous step, the texture analysis methods using statistical measurements of color channels and the BGC can be performed. The texture computation process converts image samples of the negative (pervious surface) and the positive (impervious surface) classes into numerical features. These numerical features are subsequently used for the task of pattern recognition performed by the neural computing models. The statistical measurements of the three color channels include the mean, standard deviation, skewness, kurtosis, entropy, and range indices. Since the number of the employed bands obtained from the Sentinel-2 is 3, there are 6 × 3 = 18 features attained from statistical measurements of color channels (refer to Figure 7).

**Figure 8:**
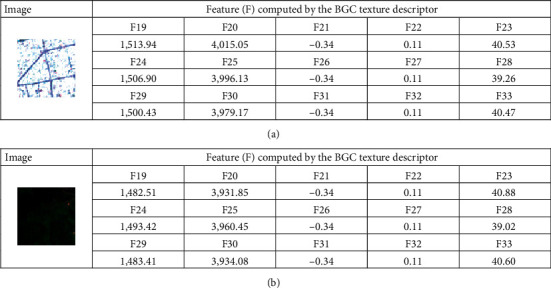
Illustration of features computed by the BGC texture descriptor: (a) an impervious surface and (b) a pervious surface.

In addition, the BGC is performed with the three versions of single-loop (BGC_1_), double-loop (BGC_2_), and triple-loop (BGC_3_). Each of them produces a histogram which describes the texture information of image samples. This study computes the measurements of mean, standard deviation, skewness, kurtosis, and entropy from each histogram. Hence, the BGC texture descriptors yield 5 × 3 = 15 features (refer to Figure 8).

Thus, the total number of features extracted from the used texture descriptors is 33. Moreover, to facilitate the data classification based on the employed neural computing model, the texture-based features have been preprocessed by the Z-score data normalization. The Z-score equation is given by

(16) $$\begin{array}{c} X_{ZN}=\frac{X_{o}-m_{X}}{s_{X}}, \end{array}$$

where *X*_*o*_ and *X*_*ZN*_ denote the original and the standardized feature, respectively; *m*_*X*_ and *s*_*X*_ represent the mean and the standard deviation of the original feature, respectively.

### 3.4. Neural Computing Model Training and Prediction

As stated earlier, a dataset including 3,000 instances and 33 features has been prepared to train and verify the neural computing approach used for impervious surface detection. Each instance of the dataset has the class label of either pervious (denoted as 0) or impervious (denoted as 1). Each data record contains texture characteristic of an image region within the map of the study area. As mentioned earlier, the statistical measurements of color channels and statistical measurements of the three BGC variants (BGC_1_, BGC_2_, and BGC_3_) are used as texture descriptors.

The neural computing model is used to generalize a decision boundary that can distinguish data instances of the two categories of pervious and impervious surfaces. Accordingly, the original dataset has been randomly split into two mutual exclusive sets: a training set (70%) and a testing set (30%). The first set is used for model construction. The latter set is used to evaluate the model's predictive capability. It is noted that the neural computing models in this study are trained with the mini-batch mode [74]. Accordingly, the training data are split into small batches and these batches are used to calculate the model error and the gradients of the neural computing models' parameters.

The training process of the neural computing model aims at adapting the two matrices of *W*_L0L1_ and *W*_L1L2_ that specify the model structure. The size of the first matrix, which is the connection weight between the input and the hidden layer, is *M* x (*N*_*I*_ + 1) matrix where *M* and *N*_*I*_ represent the number of neurons in the hidden layer and the number of input features, respectively. Herein, *N*_*I*_ = 33 which is equal to the number of features extracted from the employed texture descriptors.

It is noted that the selection of the tuning parameters of the neural computing model in this study is based on recommendation of previous works and experimental trials using the collected dataset. Based on the suggestion of Heaton [75] and Tien Bui et al. [44], the number of neurons in the hidden layer in this study is set to be (2/3)*N*_*I*_+*N*_*O*_, where *N*_*O*_ = 2 denotes the number of the output classes. In addition, the log-sigmoid is chosen as the activation function since it is commonly used for constructing shallow neural network models used for pattern classification [76, 77]. The softmax activation function is employed in the final layer to yield class probabilities within the range of 0 and 1 [67, 78]. Moreover, the number of training epochs is also required to be set appropriately. It is worth noticing that this tuning parameter may strongly affect the training outcome. An insufficient number of epochs can result in an underfitted model. Meanwhile, an excessive number of epochs may lead to an overfitted model. In this study, via several trial-and-error experiments with the collected dataset, the suitable number of training epoch is found to be 100.

When the number of neurons in the hidden layer is determined, the size of the matrices that contain connection weights can be specified. Herein, the size of the *W*_L1L2_, which stores connection weights between the hidden and output layer is *N*_*O*_*x* (*M* + 1). Thus, the total number of variables needed to be identified by the employed optimizers is *N*_*R*_*x N*_*I*_ + *N*_*O*_*x N*_*R*_ + 2. In this study, the optimizers of GDM, Adam, Adamax, Nadam, AdamW, and AMSGrad are used to search for the most appropriate values of the two matrices of *W*_L0L1_ and *W*_L1L2_.

## 4. Experimental Results

As stated earlier, to train and verify the neural computing model used for impervious surface area detection, the extracted dataset has been divided into two sets of training (70%) and testing (30%) datasets. In addition, to alleviate the undesired effect of randomness on data sampling and to accurately assess the generalization capability of the newly developed model, the training/testing data sampling processes have been performed 20 times. In each time, 30% of the dataset, which corresponds to 900 instances, is randomly drawn out to form the testing dataset. The rest of the dataset including 2100 instances is used for model training.

Based on the model configuration in the previous section, the employed neural computing model is an artificial neural network consisting of 33 neurons in the hidden layer. The log-sigmoid is used as the activation function in the hidden layer. In the output layer, the softmax function is utilized to derive the probability of the two class labels of impervious surface and pervious surface.

In addition, to evaluate the prediction results of the employed neural computing models, classification accuracy rate (CAR), precision, recall, negative predictive value (NPV), and F1 score are calculated as follows [79]:

(17) $$\begin{array}{c} \text{CAR}=\frac{\text{TP}+\text{TN}}{\text{TP}+\text{TN}+\text{FP}+\text{FN}}\times 100\%,\\ \text{precision}=\frac{\text{TP}}{\text{TP}+\text{FP}},\\ \text{recall}=\frac{\text{TP}}{\text{TP}+\text{FN}},\\ \text{NPV}=\frac{\text{TN}}{\text{TN}+\text{FN}},\\ F1\text{ score}=\frac{2\text{TP}}{2\text{TP}+\text{FP}+\text{FN}}, \end{array}$$

where TP, TN, FP, and FN represent true-positive, true-negative, false-positive, and false-negative instances, respectively.

**Figure 9:**
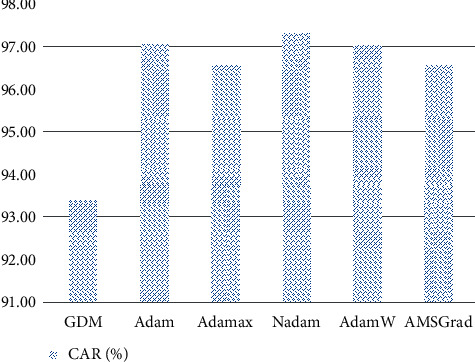
Model comparison with CAR (%).

**Figure 10:**
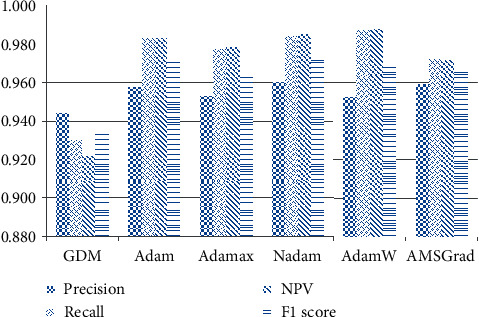
Model comparison with the indices of precision, recall, NPV, and F1 score.

The outcomes of the artificial neural network models optimized by the used optimizers obtained from the training and testing phases are reported in Tables 1 and 2. It is observable that the neural computing models optimized by the Nadam have achieved the most desired performance with CAR = 97.331%, precision = 0.961, recall = 0.984, NPV = 0.985, and F1 score = 0.972. As can be seen from Tables 1 and 2, the prediction performances obtained from the training phase (CAR = 97.967%) and testing phase (CAR = 97.311%) of the Nadam-based model are relatively close to each other. This fact indicates that the Nadam-optimized neural computing model used for impervious surface detection does not suffer from overfitting issue. The experimental results also demonstrate that the selected number of training epochs is reasonable and help to prevent both overfitting and underfitting. The Adam optimizer is the second best approach (CAR = 97.050%), followed by the AdamW (CAR = 97.028%), Adamax (CAR = 96.572%), AMSGrad (CAR = 96.556%), and GDM (CAR = 93.389%). The model result comparison is also graphically presented by Figures 9 and 10.

**Figure 11:**
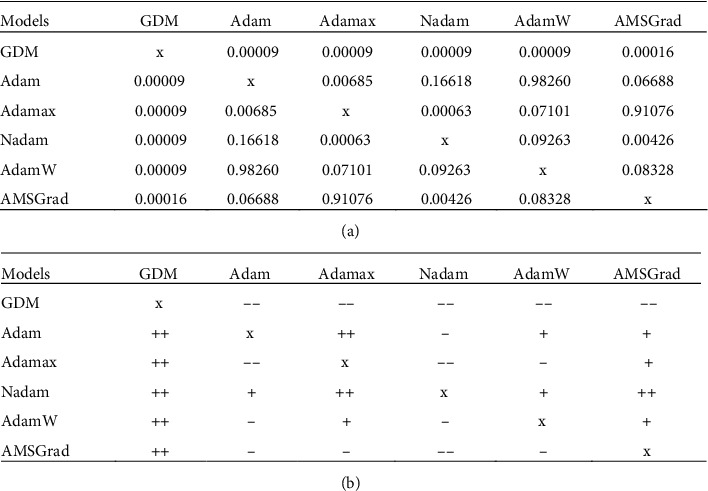
Results of the Wilcoxon signed-rank test: (a) results of p values and (b) test outcomes. *Note.* The symbols ++, +, --, and – denote a significant win, a win, a significant loss, and a loss.

**Figure 12:**
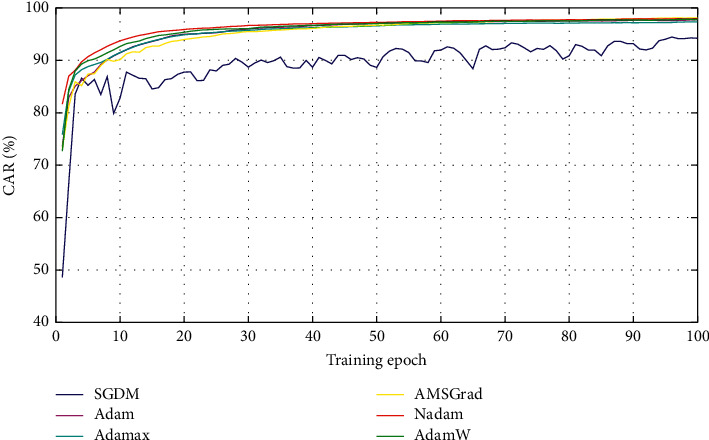
Training progress comparison.

Furthermore, to confirm the statistical difference of each pair of the neural computing models used for impervious surface detection, the Wilcoxon signed-rank test with significance level (*p* value) = 0.05 is used. The test results are provided in Figure 11. Observed from the test outcomes, all of the advanced optimizers including Adam, Adamax, Nadam, AdamW, and AMSGrad significantly outperformed the conventional GDM. The Nadam as the best approach achieves three significant wins and two wins. Notably, the benchmark method of GDM gets five significant losses. The average convergence records of all the employed optimizers are also provided in Figure 12.

The experimental results have demonstrated the superiority of the Nadam optimizer in constructing the neural computing model-based impervious surface detection for the study area. The outstanding performance of the Nadam algorithm can be explained by the fact that this advanced optimizer is a combination of the powerful Adam and Nesterov-accelerated gradient (NAG) approaches. The Adam optimizer has a significant advantage of computing adaptive learning rates for each parameter of the neural computing model [73]. Moreover, since Nesterov momentum provides a correction factor to the standard method of momentum, the NAG often results in good training performance [74]. The Nadam algorithm harnesses the advantages of the Adam and NAG approaches. Therefore, this optimizer has achieved the most desired performance for the collected dataset.

**Figure 13:**
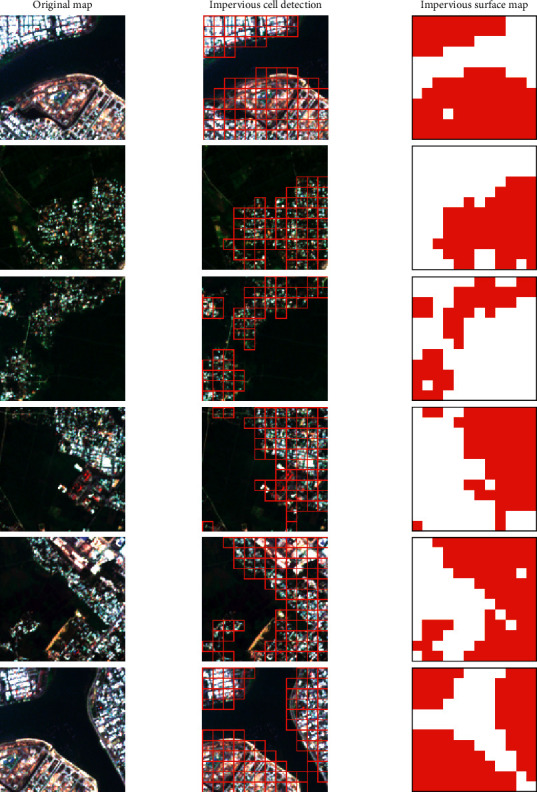
Demonstrations of the model classification outcomes with small-scale maps. *Note.* A red cell and a white cell denote impervious and pervious areas, respectively.

**Figure 14:**
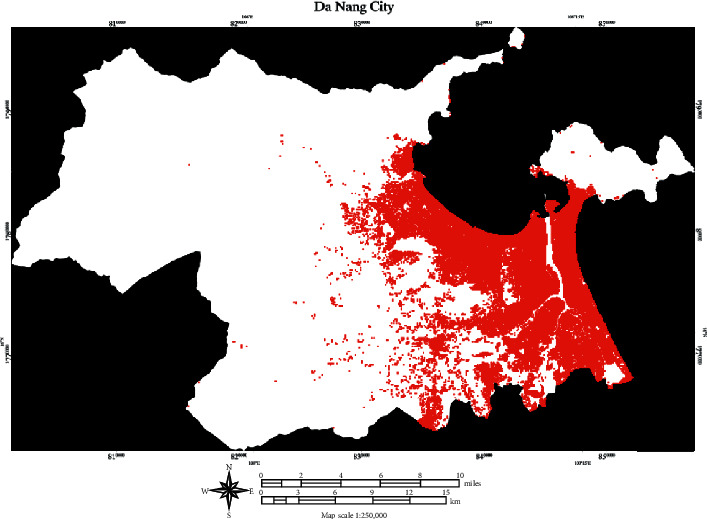
The map illustrates the impervious surface area for Da Nang city area. *Note*. A red cell and a white cell denote an impervious and pervious area, respectively.

Since the Nadam-optimized neural computing model, denoted as Nadam-NCM, has achieved an outstanding accuracy of 97.311%, this model can be employed to accomplish the objective of impervious surface mapping in a reliable manner.  Figure 13 demonstrates the application of the Nadam-based model in detecting impervious surface for small-scaled maps. The impervious surface map of the study area is provided in Figure 14. Based on the classification result, the impervious surface areas account for roughly 18.25% of the study area.

## 5. Concluding Remarks

Up-to-date information regarding the impervious surface areas is crucial for the task of land-use planning, monitoring, and management. This study investigates the employment of neural computing models trained by the advanced optimizers used for automatic impervious surface area detection. The conventional GDM algorithm and the advanced optimizers of the Adam, Adamax, Nadam, AdamW, and AMSGrad are employed to train the neural computing models used for the pattern recognition task of interest. Experimental results supported by the Wilcoxon signed-rank test points out that the Nadam-optimized neural computing model has achieved the most desired predictive accuracy with CAR = 97.311%. Therefore, this model can potentially serve as an effective tool for extracting built-up impervious surfaces at regional scale. Future extensions of the current work may include the following:

- The application of the Nadam-optimized neural computing model in impervious surface extraction for other study areas
- Investigation of capabilities of other advanced optimizers in training neural network models
- Exploring the effect of different neural network structures (e.g., different activation functions and number of neurons in the hidden layer) on the accuracy of the impervious surface detection problem
- Incorporating state-of-the-art regularization techniques (e.g., dropout regularization) into the training process of the neural computing models
- Investigating the possibility of using metaheuristic algorithms to meliorate the model training performance
- The employment of other advanced texture descriptors for improving the classification accuracy rate
- Incorporation of statistical and metaheuristic-based feature selection methods into the current model to further enhance the prediction accuracy
- Investigation of capabilities of other advanced machine learning models (e.g., deep learning and Markov models) for impervious surface extraction
- Developing intelligent models for predicting time series of remotely sensed impervious surface data with other advanced neural computing models including recurrent neural network and long short-term memory.

## Figures and Tables

**Algorithm 1:**
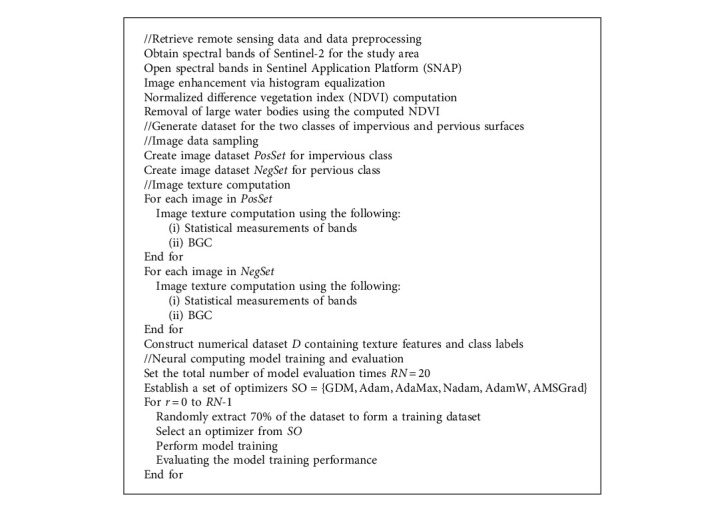
The data processing and the training phase of the proposed neural computing model used for impervious surface detection.

**Table 1:** Training performance.

Models	Statistics	Performance measurement indices								
		CAR (%)	TP	TN	FP	FN	Precision	Recall	NPV	F1 score
GDM	Mean	94.210	999.800	978.600	50.650	70.950	0.952	0.939	0.933	0.944
	Std	3.613	28.037	82.229	31.922	86.497	0.030	0.060	0.081	0.030

Adam	Mean	97.771	1,008.450	1,044.750	36.850	9.950	0.965	0.990	0.991	0.977
	Std	0.348	11.859	12.825	7.164	3.186	0.007	0.003	0.003	0.004

Adamax	Mean	97.340	1,008.500	1,035.650	42.700	13.150	0.959	0.987	0.987	0.973
	Std	0.392	18.629	15.415	5.780	3.991	0.006	0.004	0.004	0.004

Nadam	Mean	97.967	1,019.050	1,038.250	36.250	6.450	0.966	0.994	0.994	0.979
	Std	0.367	14.116	13.634	6.898	2.636	0.006	0.003	0.003	0.004

AdamW	Mean	97.793	1,014.100	1,039.550	40.050	6.300	0.962	0.994	0.994	0.978
	Std	0.310	12.992	12.698	6.289	3.480	0.006	0.003	0.003	0.003

AMSGrad	Mean	98.117	1,016.450	1,044.000	29.250	10.300	0.972	0.990	0.990	0.981
	Std	0.465	13.959	14.601	7.175	5.178	0.007	0.005	0.005	0.005

**Table 2:** Testing performance.

Models	Statistics	Performance measurement indices								
		CAR (%)	TP	TN	FP	FN	Precision	Recall	NPV	F1 score
GDM	Mean	93.389	424.700	415.800	24.850	34.650	0.944	0.930	0.922	0.935
	Std	3.428	23.046	41.248	15.203	35.373	0.035	0.055	0.081	0.027

Adam	Mean	97.050	435.550	437.900	19.150	7.400	0.958	0.983	0.983	0.970
	Std	0.522	11.182	11.388	4.542	3.216	0.010	0.007	0.007	0.005

Adamax	Mean	96.572	427.800	441.350	21.000	9.850	0.953	0.977	0.978	0.965
	Std	0.728	17.189	16.487	6.025	2.903	0.013	0.007	0.006	0.008

Nadam	Mean	97.311	427.250	448.550	17.450	6.750	0.961	0.984	0.985	0.972
	Std	0.420	12.980	11.923	4.444	3.064	0.010	0.007	0.006	0.005

AdamW	Mean	97.028	424.600	448.650	21.250	5.500	0.952	0.987	0.988	0.969
	Std	0.520	11.061	11.897	4.898	2.110	0.011	0.005	0.005	0.005

AMSGrad	Mean	96.556	435.800	433.200	18.500	12.500	0.959	0.972	0.972	0.966
	Std	0.840	12.659	14.020	4.955	5.005	0.011	0.011	0.011	0.008

## Data Availability

The dataset used to support the findings of this study has been deposited in the repository of GitHub (https://github.com/NhatDucHoang/NCM_ISD_DaNang).
